# Structural and Dynamic Characterization of the C313Y Mutation in Myostatin Dimeric Protein, Responsible for the “Double Muscle” Phenotype in Piedmontese Cattle

**DOI:** 10.3389/fgene.2016.00014

**Published:** 2016-02-11

**Authors:** Silvia Bongiorni, Alessio Valentini, Giovanni Chillemi

**Affiliations:** ^1^Department for Innovation in Biological, Agro-food and Forest systems, University of TusciaViterbo, Italy; ^2^Department of SuperComputing Applications and Innovation, CinecaRome, Italy

**Keywords:** Myostatin, Piedmontese mutation, C313Y, double muscling, molecular dynamics, dimer asymmetry

## Abstract

The knowledge of the molecular effects of the C313Y mutation, responsible for the “double muscle” phenotype in Piedmontese cattle, can help understanding the actual mechanism of phenotype determination and paves the route for a better modulation of the positive effects of this economic important phenotype in the beef industry, while minimizing the negative side effects, now inevitably intersected. The structure and dynamic behavior of the active dimeric form of Myostatin in cattle was analyzed by means of three state-of-the-art Molecular Dynamics simulations, 200-ns long, of wild-type and C313Y mutants. Our results highlight a role for the conserved Arg333 in establishing a network of short and long range interactions between the two monomers in the wild-type protein that is destroyed upon the C313Y mutation even in a single monomer. Furthermore, the native protein shows an asymmetry in residue fluctuation that is absent in the double monomer mutant. Time window analysis on further 200-ns of simulation demonstrates that this is a characteristic behavior of the protein, likely dependent on long range communications between monomers. The same behavior, in fact, has already been observed in other mutated dimers. Finally, the mutation does not produce alterations in the secondary structure elements that compose the characteristic TGF-β cystine-knot motif.

## Introduction

**FIGURE 1:**
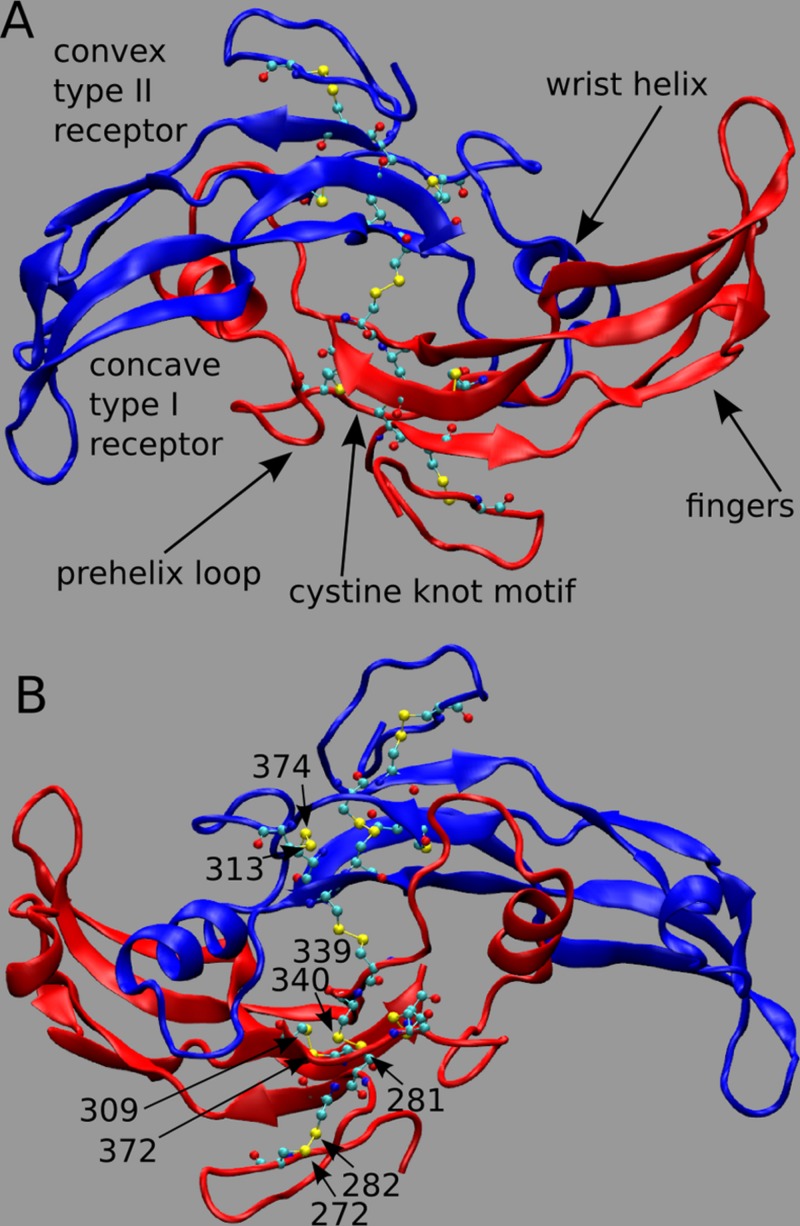
**(A,B)** 3D structure of myostatin (MSTN) in dimeric form. The two dimers are in red and blue colors. Each monomer is composed by four curved beta strands (fingers), an alpha-helix (wrist helix), a pre-helix loop, and a cystine-knot motif composed by disulfide bonds between residues 272–282; 281–340; 309–372; and 313–374. Moreover an inter-chain disulfide bond between residues Cys339 links the two monomers. The active dimer presents two convex type II receptor-binding sites and two concave type I receptor-binding sites (indicated as convex and concave, respectively). Piedmontese-derived MSTN mutation C313Y eliminates the disulfide bond 313–374.

*Myostatin* (*MSTN*), also named growth differentiation factor-8, is a member of the transforming growth factor-beta (TGF-beta) superfamily and is the primary negative regulator of skeletal muscle development (Beyer et al., 2013). MSTN circulates in the blood as a full-length precursor, which is cleaved into a N-terminal pro-peptide and a C-terminal mature region (Hill et al., 2002; Lee, 2008). In **Figure 1**, we show the functional state of MSTN, composed by two C-terminal monomers (residue 267–375) linked by an inter-chain disulfide bond between residues Cys339 (**Figure 1A**; PDB id: 3HH2; Cash et al., 2009). MSTN is also characterized by four intra-monomer disulfide bonds between cysteines 272–282; 281–340; 309–372; and 313–374. These nine highly conserved cysteine residues are typical of the members of the TGF-β superfamily (McPherron et al., 1997), and together with the inter-chain disulfide bond form the characteristic TGF-β cystine-knot structural motif.

*Myostatin* signaling acts through the activin receptor type IIA (ActRIIA) or ActR-IIB and either TβRI/ALK-5 or ALK-4, type I receptors on skeletal muscle, triggering the activation of TGF-β–specific *Smads, Smad2* and *Smad3* followed by oligomerization with *Smad4* (Massagué and Wotton, 2000). The Smad protein complex translocates into the nucleus, where it regulates transcription of specific myogenic regulatory genes such as Myod (Langley et al., 2002). Inhibition of this pathway results in muscle hyperplasia (Lee and McPherron, 2001; Lee, 2007). Smad7 has been shown to inhibit both TGF-β1 and MSTN signaling, and to enhance skeletal muscle differentiation (Kollias et al., 2006). MSTN-Propeptide exhibits high binding affinity for MSTN, and it has been shown to be a potent inhibitor of MSTN. Antagonists of MSTN activity such as the follistatin which hinders access to signaling receptors on skeletal muscle (Sumitomo et al., 1995), are considered as potential therapeutics in the treatment of muscle-wasting disorders such as muscular dystrophy and sarcopenia (Bogdanovich et al., 2002, 2005).

Myostatin dimer activity can be inhibited by non-covalent binding of two monomeric MSTN pro-peptides (self-regulation) with each binding a concave type I receptor-binding site (in the sheet or “finger” region) and a convex type II receptor-binding site (composed of the “fingertip” and the “wrist” helix, see **Figure 1A**; Lee and McPherron, 2001; Yang et al., 2001).

*Myostatin* gene is highly conserved among vertebrate species and knockout mouse line for the *MSTN* gene shows a significant increase in skeletal muscle mass. The so-called “double muscling” phenotype has been observed in different animal species such as dogs (Mosher et al., 2007), sheep (Clop et al., 2006; Kijas et al., 2007), cattle (Grobet et al., 1997), pigs (Stinckens et al., 2008), and human (Schuelke et al., 2004). In cattle several different breeds harbor mutations in *MSTN* gene and show a hereditary muscular hyperplasia (double-muscled cattle): Belgian Blue, Piedmontese, Charolais, Limousin, Ford, Holstein-Friesian, Angus, Marchigiana, Maine-Anjou, Blonde d’Aquitaine, Parthenaise, Gasconne, Asturiana de los Valles, and Rubia Gallega (**Table 1**).

**Table 1:** Summary of major mutations in the myostatin gene.

Mutation name	Change at gene level	Change at protein level	Breed	Reference
nt821	Deletion of 11 bp at nucleotide position 821	Truncated protein due to a premature STOP codon in the bioactive C-terminal domain	Belgian Blue, Blonde d’Aquitaine, Limousin, Parthenaise, Asturiana de Valles, Rubia Gallega	Dunner et al., 1997; Grobet et al., 1997, 1998; Kambadur et al., 1997; McPherron and Lee, 1997; Allais et al., 2010
C313Y	G→A transition at nucleotide position 938	Substitution of a highly conserved cysteine involved in a intramolecular disulfite bridge in the bioactive C-terminal domain, by a tyrosine	Piedmontese, Gasconne	McPherron and Lee, 1997; Libor, 2008
nt419 (del7- ins10)	Insertion/deletion at nucleotide position 419	truncated protein due to a premature STOP codon in the N-terminal latency-associated peptide	Maine-Anjou	Grobet et al., 1998
Q204X	C→T Transition at nucleotide position 610	Truncated protein due to a premature STOP codon in the N-terminal latency-associated peptide	Charolais, Limousin	Allais et al., 2010
E226X	G→T Transversion at nucleotide position 676	Truncated protein due to a premature STOP codon in the N-terminal latency-associated peptide	Maine-Anjou	Grobet et al., 1998
E291X	G→T Transversion at nucleotide position 874	Truncated protein due to a premature STOP codon in the bioactive C-terminal domain	Marchigiana	Cappuccio et al., 1998; Marchitelli et al., 2003
T–371 > A–371 G–805 > C-805	T→A Transversion at nucleotide position –371; G→C transversion at nucleotide position –805	Promoter	Marchigiana, Chianina, Romagnola, Piedmontese, Holstein Friesian, Italian Red Pied, Brown Swiss, Belgian Blue, Limousine	Crisà et al., 2003
G-7828 > C-7828	G→C Transversion at position -7828	5′-Flanking region	Holstein-Friesian	Sadkowski et al., 2008
T3811 > G3811	Intronic mutation	An abnormal transcript with a premature termination codon	Blonde d’Aquitaine	Bouyer et al., 2014

All these mutations, located in the bioactive carboxyl-terminal domain, result in an impairment of *MSTN* function and promote muscle growth (McPherron and Lee, 1997). However, the most powerful mutations are those affecting the highly conserved cysteine residues (Cash et al., 2009). In particular, the deletion in Belgian Blue introduces a frame shift and a stop codon, while in Piedmontese a “simple” transition G→A at nucleotide position 938 results in the substitution of a cysteine by a tyrosine (C313Y; Kambadur et al., 1997), thus eliminating one of the disulfide bond (313–374) that is part of the TGF-β cystine-knot structural motif. At the best of our knowledge the destabilizing effect of this mutation has not yet been investigated by a molecular point of view.

Molecular dynamics (MD) simulation is a powerful tool for examining structural and dynamic properties of biological macromolecules since it provides a description at atomic level and at the appropriate time scale. Comparing MD of native and mutant proteins, in particular, can efficiently highlight the perturbation effect of single residue mutations (Chillemi et al., 2005, 2008).

In the present paper, we aim at studying how the substitution C313Y could affect the structure and function of MSTN. We will report three 200-ns long MD simulations of the MSTN dimer (1) in the native form; (2) in the mutant, which lack the 313–374 disulfide bond in the bioactive carboxyl-terminal peptide of both monomers (i.e., The “Piedmontese mutation”); (3) in the heterodimer with the mutation only in one monomer. We focused on this mutation because of the simplicity of the model that allows for a precise understanding of the mechanism of a single aminoacidic substitution. Moreover, we hoped to clarify how such apparently minimal difference could explain a large phenotypic variation between wild-type and homozygous mutant individuals.

Our results indicate that the mutation does not alter the local structure of the protein, while it affects its dynamical properties far from the mutation site.

## Materials and Methods

### Model Generation and Simulation Protocol

Atomic coordinates of MSTN in the active dimeric state were obtained from protein data bank (id: 3HH2; Cash et al., 2009). Note that the residue numbering in the PDB file is 1–109, corresponding to residue 267–375. The starting model was built with the gromacs pdb2gmx tool (Pronk et al., 2013) and modeling the four cysteine disulfide bonds between residues 272–282; 281–340; 309–372; and 313–374 in each monomer, plus the inter-monomer disulfide bond between Cys339. Two additional models were built introducing the Piedmontese-derived MSTN mutation C313Y (Grobet et al., 1997; Kambadur et al., 1997) in one (1-copy) or both monomers (2-copy), thus eliminating the 313–374 disulfide bond.

The starting structures were embedded in a dodecahedron box, extending up to 12 Å from the solute, and immersed in TIP3P water molecules (Jorgensen et al., 1983). Counter ions were added to neutralize the overall charge with the genion gromacs tool. After energy minimizations, the systems were slowly relaxed for 5 ns by applying positional restraints of 1000 kJ mol^-1^ nm^-2^ to the protein atoms. Then unrestrained MD simulations were performed from the final structures of the restrained runs for a length of 200 ns with a time step of 2 fs (i.e., for 100,000,000 steps). V-rescale temperature coupling was employed to keep the temperature constant at 300 K (Bussi et al., 2007). All the MD simulations were performed with the Gromacs 4.5.6 package (Pronk et al., 2013) and the amber99sb-ildn force field (Lindorff-Larsen et al., 2010). The Particle-Mesh Ewald method was used for the treatment of the long-range electrostatic interactions (Darden et al., 1993). Only the 0-copy and 2-copy systems were simulated for further 200 ns in order to investigate the RMSF asymmetry of the two monomers in the first system but not in the second one.

### Molecular Dynamics Analyses

The dynamic cross-correlation (DCC) map (McCammon and Harvey, 1988) was built with the gromacs g_covar tool. Per-residue RMSF, hydrogen bonds and secondary structure content were obtained with the gromacs tools g_rmsf, g_hbond and do_dssp, which is an interface to the DSSP program (Kabsch and Sander, 1983). The figures in the 3D structures were generated with vmd (Humphrey et al., 1996).

## Results

The structural and dynamic effect of the C313Y mutation on the MSTN protein has been investigated in the dimeric form, i.e., the functional state of MSTN, with the following models: (1) the wild-type form (0-copy); the heterodimer composed by the monomer in mutated form and the second in wild-type one (1-copy); (2) the homodimer mutant (2-copy).

The root mean square deviation (RMSD) plot as a function of simulation time is usually used to check the protein stability during the simulation. An unfolding protein, in fact, has a RMSD always growing. The RMSD plot of all the three simulated systems (Supplementary Figure S1) is very stable for the whole time window, therefore telling us that the system has reached a potential energy minimum and is sampling the available conformational space.

In line, the secondary structure of both monomers, i.e., the β strands forming the fingers and the wrist helix (**Figure 1**), are conserved in both wild-type and mutant systems during the whole simulation (Supplementary Figure S2).

Therefore our results indicate that the loss of the 313–374 disulfide bond in one or both monomers, is not enough to destroy the highly stable cysteine-knot structure. A compensative structural effect, in particular, is likely performed by the 309–372 disulfide bond.

**FIGURE 2:**
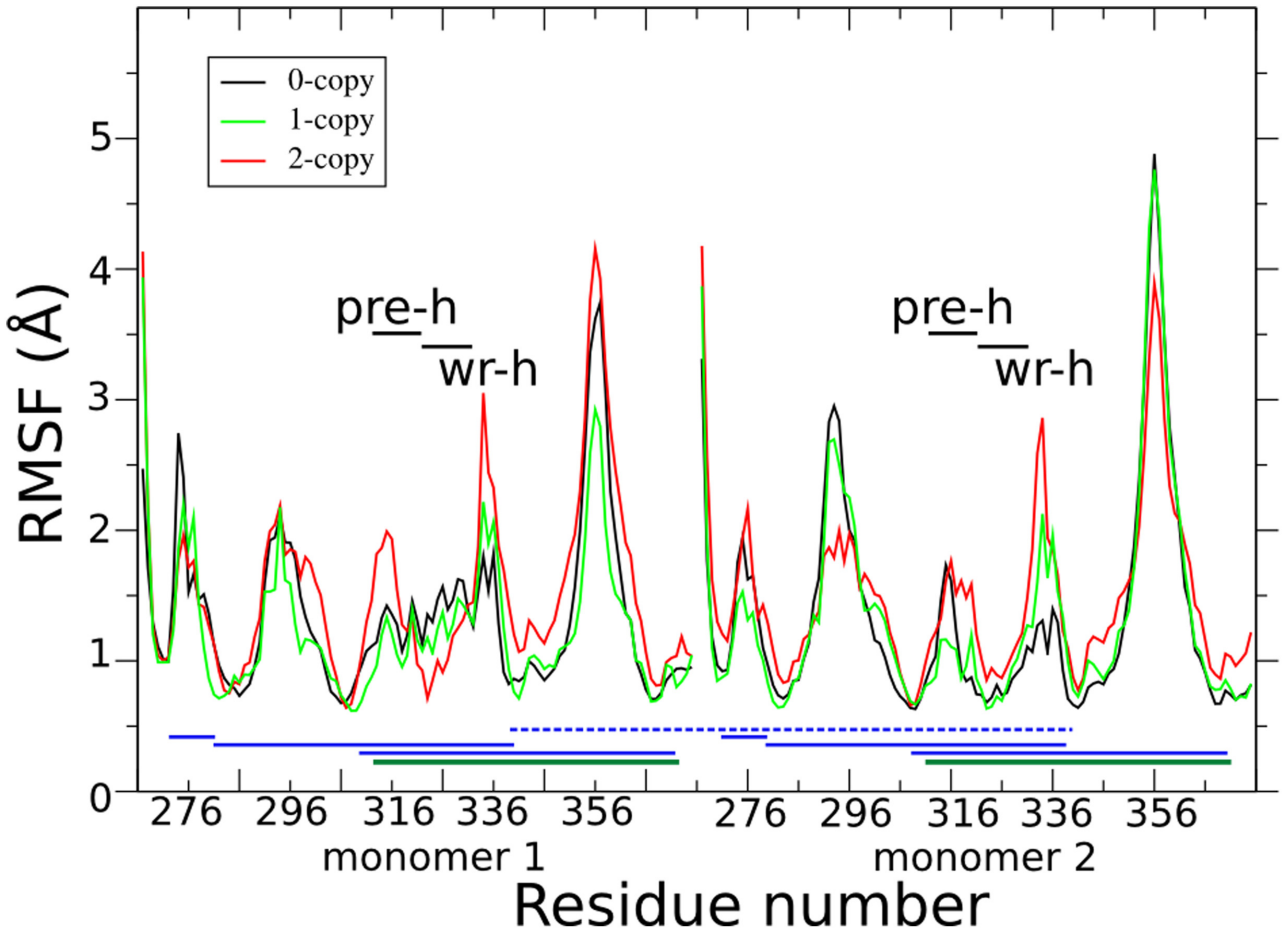
**Per-residue root mean square fluctuations (RMSF) are shown in black, green, and red lines for the 0-, 1-, and 2-copy dimers, respectively.** Horizontal black lines indicate the pre-helix and wrist helix regions. Blue horizontal lines link the cysteine residues connected by the disulfide bonds in each monomer. The dark green horizontal lines highlight the disulfide bond between Cys313 and Cys374, abolished by the C313Y mutation. The blue horizontal dashed line connects the inter-monomer Cys339 disulfide bond.

**Figure 2** shows the per-residue root mean square fluctuations (RMSF) in black, green, and red lines for 0-, 1-, and 2-copy mutations, respectively. The loop corresponding to the 354–358 residues is always the most fluctuating region. Note that this loop forms part of the concave type I receptor-binding site (**Figure 1A**). The 2-copy system shows a significant greater fluctuation in the 332–337 residues, at the C-term of the wrist helix. The 0-copy system is quite stable in this region, while the 1-copy shows an intermediate behavior. It is quite interesting that the greatest RMSF differences between mutant and native systems are not located close to the mutation site.

Comparison of fluctuations between the two monomers in each of the three systems (Supplementary Figure S3) shows that both the 0- and 1-copy systems have an asymmetric fluctuation of their monomers, while the RMSF profile in the 2-copy system is very similar in both monomers. It is likely to assume that the asymmetric fluctuations in 1-copy system is due to the presence of the mutation in only one of the two monomers. The RMSF differences between 0- and 2-copy systems are further discussed in the following sections, after the long range interaction and hydrogen bond analyses.

**FIGURE 3:**
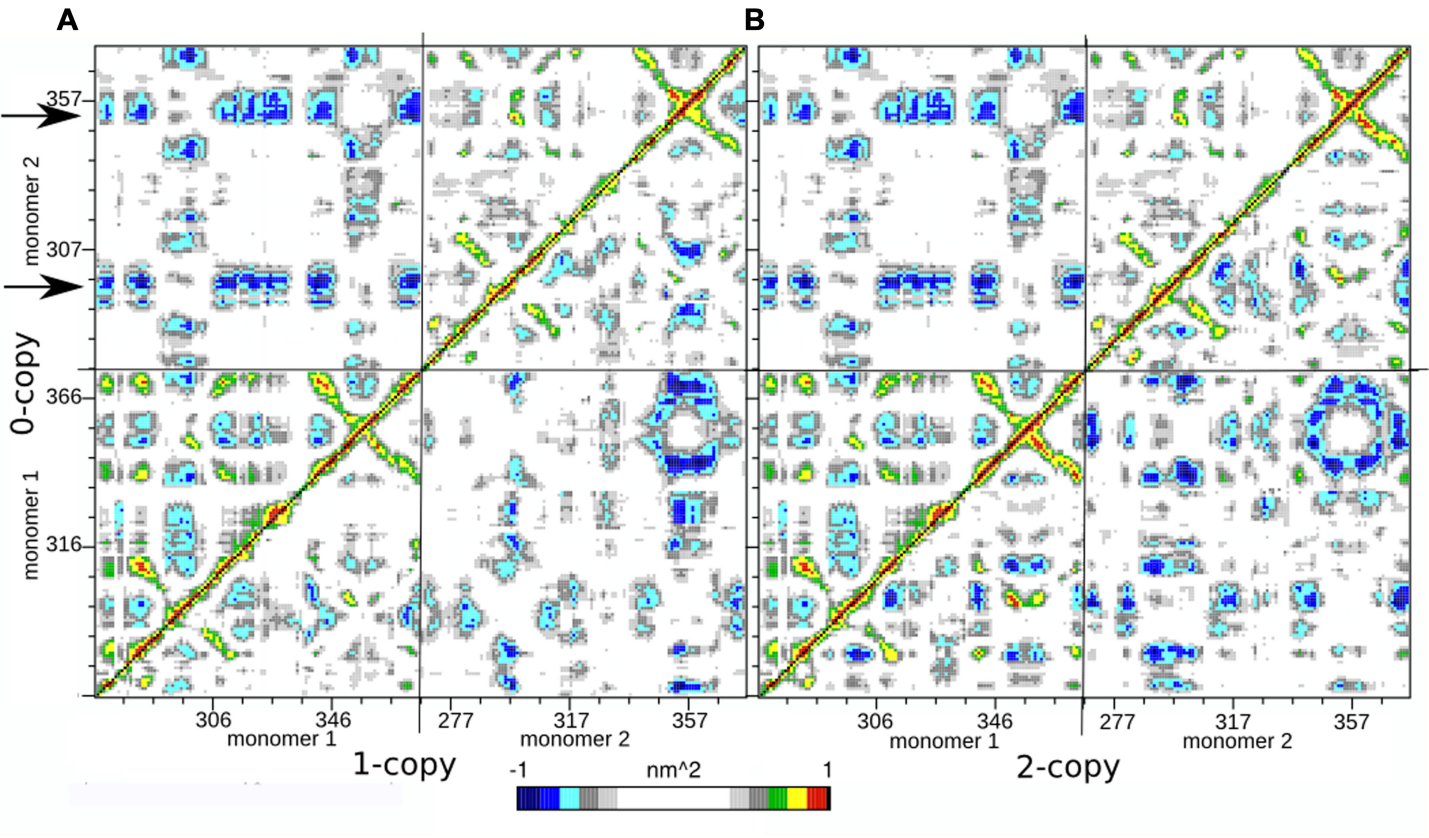
**Dynamic Cross Correlation Map (DCCM). (A)** Comparison of 0- and 1-copy system is reported in the upper left and lower right triangles, respectively. **(B)** Comparison of 0- and 2-copy system is reported in the upper left and lower right triangles, respectively.

In order to further investigate the long range interactions in these systems, we have built the Dynamic Cross Correlation Maps (DCCMs) and reported the comparison between the three systems in **Figure 2**. This analysis gives an overall picture of the correlated motions that occur between protein residues during the simulation. Highly positive peaks of the elements of the map (Cij) are indicative of a strong correlation between the movement of residues i and j (colored in green, yellow, and red in **Figure 3**); the diagonal of each DCCM is black because each residue has a correlation of 1 with itself; negative Cij values denote that the two residues move in opposite directions (anti-correlated motion; colored in cyan, light, and dark blue in **Figure 3**). Both positive correlation than anti-correlation movements are relevant when investigating biological macromolecules, particularly in couples of residues that are located far apart in the 3D structure.

Since each map is symmetrical, we combined two DCCMs in one as in the following: the comparison between 0-copy (upper left triangle) and 1-copy dimers (lower right triangle) is reported in **Figure 3A**; the comparison between 0-copy and 2-copy dimers (lower right triangle) is reported in **Figure 3B**. The most striking difference between native and mutant systems is in the anti-correlated motions between monomer 1 and 2. Two specific regions in the 0-copy system, composed by residues 288–298 and 351–359 in monomer 2 and highlighted by two horizontal arrows, are strongly anti-correlated with several regions of monomer 1. The same two regions in monomer 1 show an analogous anti-correlated motion with several regions of monomer 1 but with reduced intensity.

These coordinated motions of monomer 1 with monomer 2 are lost in both the mutant systems. Both 1-copy and 2-copy, in fact, show a monomer 1-monomer 2 anti-correlation motion concentrated only between the respective finger regions (residues 80–105), while the remaining portions of the protein do not show significant correlations in their movements.

**FIGURE 4:**
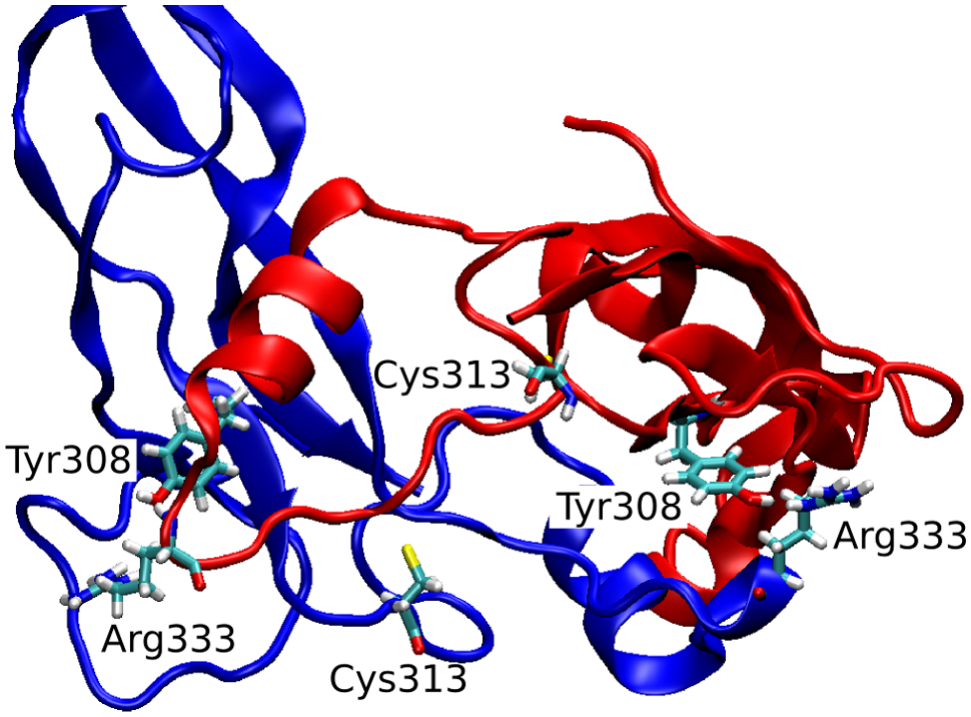
**3D snapshot of the inter-monomeric interactions between Arg333 and Tyr308**.

The observed great perturbation in inter-monomer communications can be further analyzed by the hydrogen bond analysis. In **Table 2**, the hydrogen bonds with residence time greater than 40% of simulation time are reported. It is worth noting that four out of five long residence hydrogen bonds in the wild-type system involve Arg333, at the C-term of the wrist helix and very well conserved among mammals. The interaction between Arg333 and Tyr308 of the other monomer, in particular, is very stable and present in both monomers of the copy-0 system. A snapshot of the MD simulation with the two highlighted residues is shown in **Figure 4**.

**Table 2:** Inter-monomer hydrogen bonds with residence time greater than 40% of simulation time (in bold residues belonging to monomer 2).

	Donor	Acceptor	% Residence time
0-copy	TYR308 OH	**ARG333 O**	58.3
	ARG333 NE	**TYR308 OH**	94.6
	ARG333 NH1	**ASP273 O**	40.9
	ARG333 NH2	GLU274 OE1	82.4
	GLY334 N	TYR308 OH	42.8
1-copy	GLN329 NE2	**GLU291 OE2**	52.7
	GLN329 NE2	**ALA292 O**	88.5
	GLN329 NE2	**PHE293 O**	50.1
	**ARG333 NH1**	GLU274 OE2	46.6
	**ARG333 NH2**	TYR308 OH	41.7
	**THR341 OG1**	SER375 OC2	43.5
2-copy	ARG333 NH1	**GLU274 O**	91.5
	**ARG333 NH1**	SER276 O	90.6
	**ARG333 NH1**	TYR284 OH	47.4

The C317Y mutation strongly perturbs the h-bond network observed in the copy-0 system. The Arg333-Tyr308 interaction, in fact, is observed in the 1-copy-dimer only between the native monomer 2 and the mutant monomer 1; and it is completely lost in the 2-copy system, with Arg333 interacting only with the N-terminal region of the corresponding other monomer.

The observed RMSF asymmetry between the two monomers of 0-copy are nearly abolished in the 2-copy system (**Figure 2** and Supplementary Figure S3). Furthermore, we carried out two simulations of these two systems, each 200 ns long and independent from the previously described. Per-residue RMSF analysis of four time windows, each 50 ns long, is shown in Supplementary Figure S4 as box-plot data for monomer 1 and 2 (blue and green colors, respectively) and for 0-copy and 2-copy (panel A and B, respectively). An asymmetric behavior is confirmed in the 0-copy system, particularly in the 296–302, 314–317, and 325–337 residue ranges. In 2-copy system, on the contrary, limited asymmetric RMSFs are observed and only in the 315–322 residue range.

## Discussion

The *MSTN* gene, transcripts and protein, has been extensively studied in many species. In livestock, in particular, this has been one of the first genes recognized having an economic importance and has attracted a considerable attention from the scientific community.

To our knowledge, this is the first attempt to model the active dimer form of MSTN. In particular, we describe the behavior of parts of the molecule when composed by the wild-type or one of the existing mutations, or the combination of both. Understanding the MD features of normal, mutant and heterodimer MSTN provides a scientific basis for understanding the molecular reasons at the basis of the double muscling phenotype and the phenotypic effect of the mutation.

Cys313 is located in the pre-helix loop, a region known to be important for type I receptor specificity (Nickel et al., 2005; Cash et al., 2009, 2012; Kotzsch et al., 2009). Our *in silico* results indicate that the effect of the Piedmontese C313Y mutation on the 3D structure of the active MSTN dimer is quite subtle, probably due to the 309–372 disulfide bond that maintains the structure of the dimer even in the absence of the 313–374 disulfide bond. The greatest differences between native and mutant systems are in the dynamics of the single monomers and in the communications between monomers. The region 332–337 shows a significant increase in fluctuations (**Figure 2**), particularly for the 2-copy system but it is observed also in 1-copy. In line, Arg333, located in this region, forms very stable hydrogen bonds with the opposite monomer in the 0-copy dimer (**Table 2**), in particular with Tyr308. In copy-1 the Arg333-Tyr308 hydrogen bond is maintained only between Arg333 in the native monomer 2, and Tyr308 in the mutant monomer 1; while both inter-monomer interactions are lost in the 2-copy system.

The presence of these two symmetric bonds between Arg333 and Tyr308 can explain the greater anti-correlation motion observed between the two monomers in the native 0-copy dimer (**Figure 3**). The complex network of anti-correlated motions shown by the wild-type system, in fact, is lost in both 1-copy and 2-copy mutant systems, where only the finger region (346–371) of monomer 1 shows an anti-correlated motion with the same region in monomer 2.

The RMSF asymmetry; the presence of strong anti-correlation motion between monomers; and the h-bond network in the wild type enzyme, therefore, are indicative of a functional cooperative mechanism in the wild type protein that is lost upon C313Y mutation. Actually functional communications between monomers, linked to an asymmetric behavior, have been already observed in Superoxide Dismutase (Falconi et al., 1996; Chillemi et al., 1997), together with their perturbations upon mutations (Falconi et al., 1999). A functional break of the tetramer symmetry has been also recently observed in the p53-DNA complex (D’Abramo et al., 2015).

In Piedmontese, *MSTN* mutation (a missense mutation in exon 3) has been found partially recessive with heterozygotes showing a muscle mass intermediate but closer to that of wild-types (McPherron and Lee, 1997; Georges, 2010). At least for the Piedmontese mutation, we can hypothesize that of the possible dimers that form the active MSTN in the heterozygotes only one out of four are strongly affected. In fact, calling N the normal monomer and M the mutant one, the possible combinations yielding the dimer in the heterozygote are NN, NM, MN, and MM. Only the last one will show the strongly deviated behavior. Therefore, most of the circulating MSTN dimers behave “normally.” It could be reasonable to extend this hypothesis to all other mutations, i.e., that only one out of four possible dimers is affected in the heterozygotes, thus explaining the phenotypic observations (Wiener et al., 2002), however, a specific dynamic simulation for each mutation will be necessary to substantiate the hypothesis.

Homozygote double muscling mutant cattle are more susceptible to genetic disorders such as arthrogryposis (Anderson et al., 2008; Fiems, 2012), while several studies support the notion that a single copy of the mutant allele has relatively large effects on carcass characteristics, without a negative effect on calving, compared with no copies of the allele (Arthur, 1995; Casas et al., 1998).

Our study aimed at naturally occurring mutations but recent technological advancements in genome engineering, such as the cloning of cattle by somatic cell nuclear transfer or chromatin transfer, offers some extraordinary possibilities to the beef industry (Wang, 2015). Therefore, we can foresee that in the near future an animal will be edited to have the very best variants its species can offer, by natural variation or induced one where permitted (http://www.fda.gov/AnimalVeterinary/DevelopmentApprovalProcess/GeneticEngineering/GeneticallyEngineeredAnimals/ucm466214.htm).

In this framework, our study is a first step toward a full understanding of the “double muscle” phenotype within the molecular level, that in a close future can allow the beef industry to fully take advantage of the positive characteristics of this phenotype, without its negative side effects

## Author Contributions

AV and GC conceived and designed the work; all authors analyzed, interpreted data, and wrote the manuscript.

## Conflict of Interest Statement

The authors declare that the research was conducted in the absence of any commercial or financial relationships that could be construed as a potential conflict of interest.
