# Supplementary material for: Structural and Dynamic Characterization of the C313Y Mutation in Myostatin Dimeric Protein, Responsible for the “Double Muscle” Phenotype in Piedmontese Cattle
Source: Front Genet. 2016 Feb 11;7:14. doi: 10.3389/fgene.2016.00014 (PMC4749705; doi:10.3389/fgene.2016.00014)
Supplement: Supplementary file 1 [file Presentation_1.PDF]

**Supplementary Figures**

# Structural and dynamic characterization of the C313Y mutation in Myostatin dimeric protein, responsible for the "double muscle" phenotype in Piedmontese cattle

Silvia Bongiorni, Alessio Valentini, and Giovanni Chillemi*

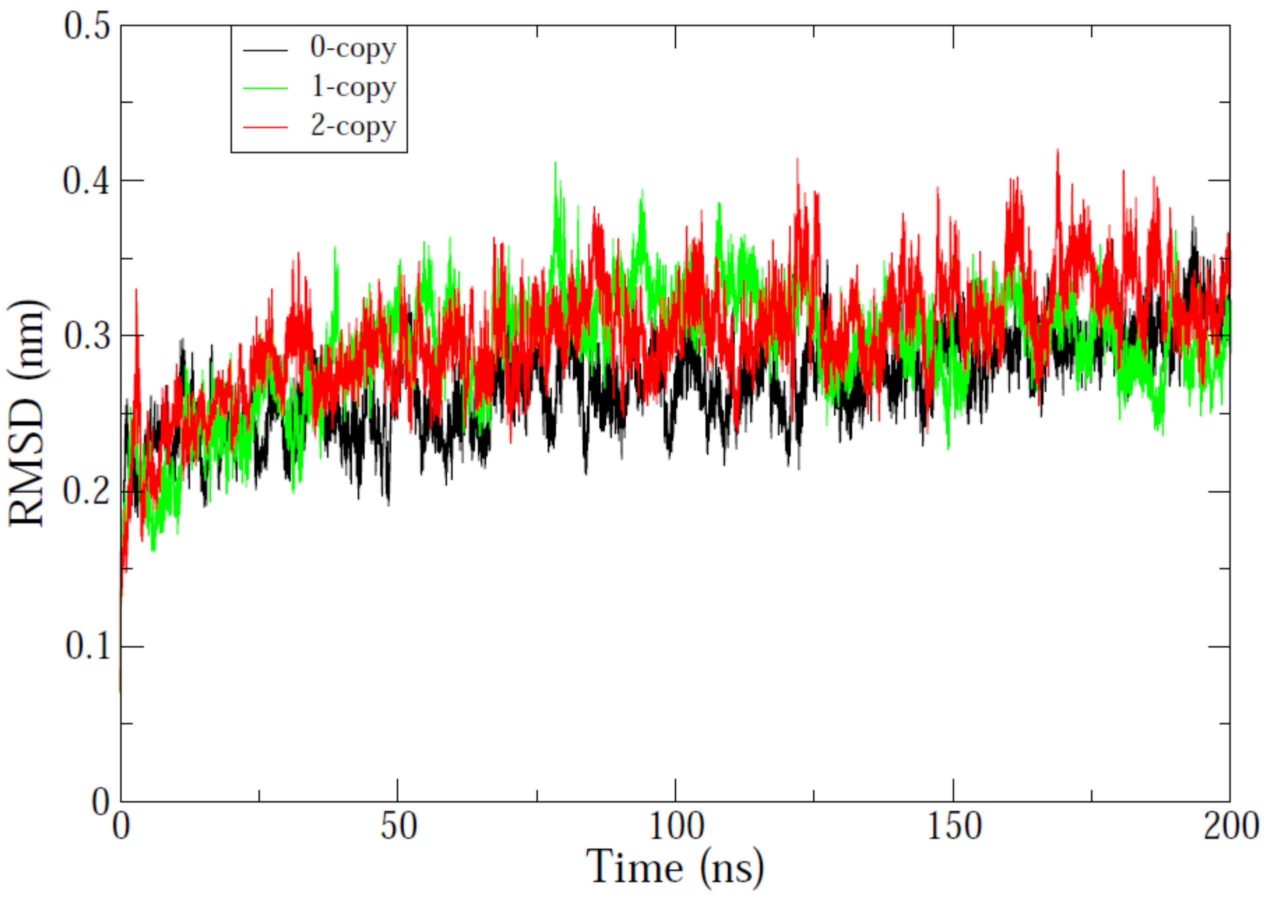


**Figure S1** Root mean square deviation (RMSD) of protein backbone as a function of simulation time is shown in black, green and red lines for the three simulated systems: 0-, 1- and 2-copy mutations, respectively. All the systems are quite stable and the maximum deviation from the starting X-ray structure is less than 4 Å.

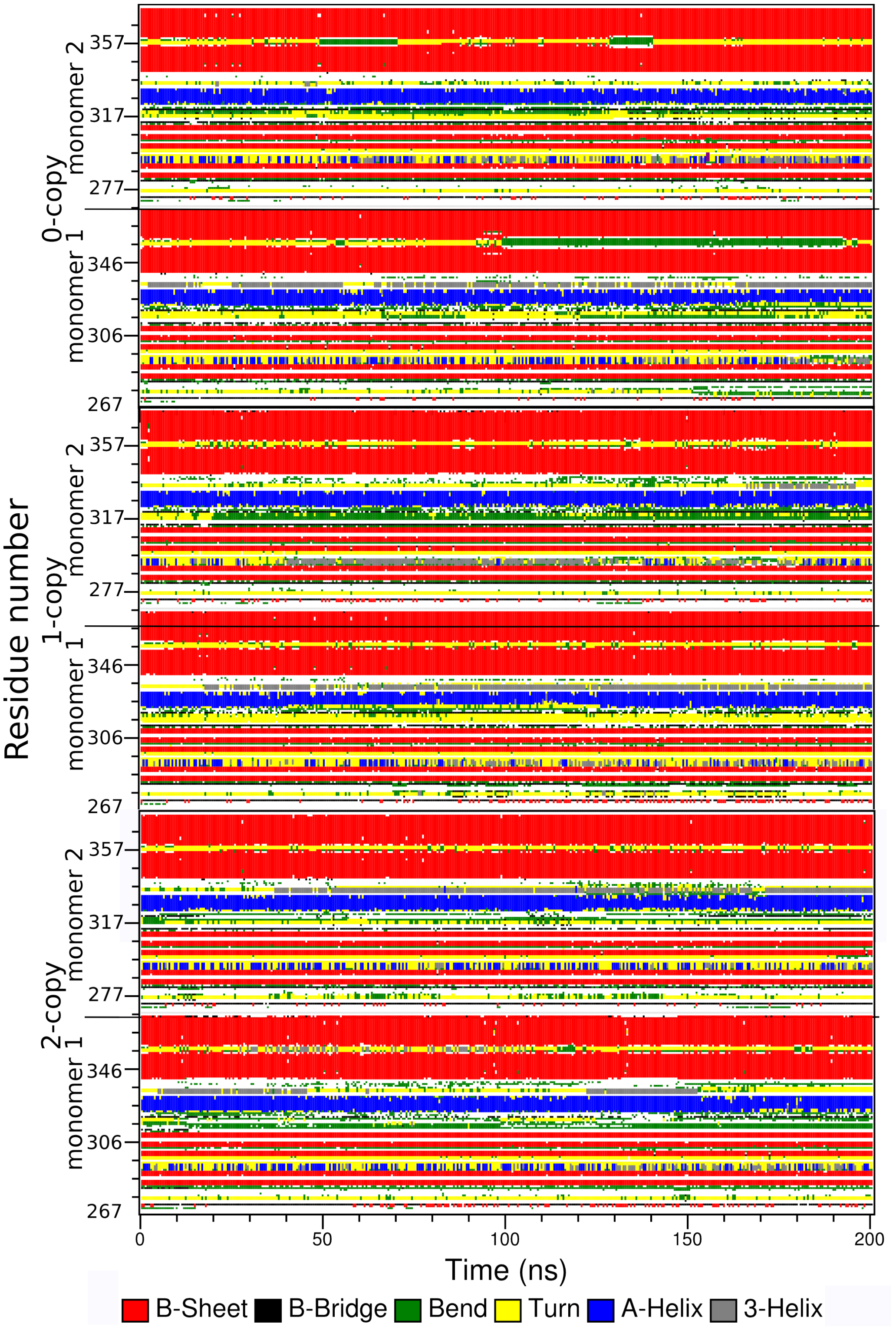


**Figure S2.** Secondary structure content as a function of simulation time is shown for the 0-, 1-, and 2-copy dimers. All the beta strands and the wrist helix are well conserved over the whole simulation length, in all the three systems.

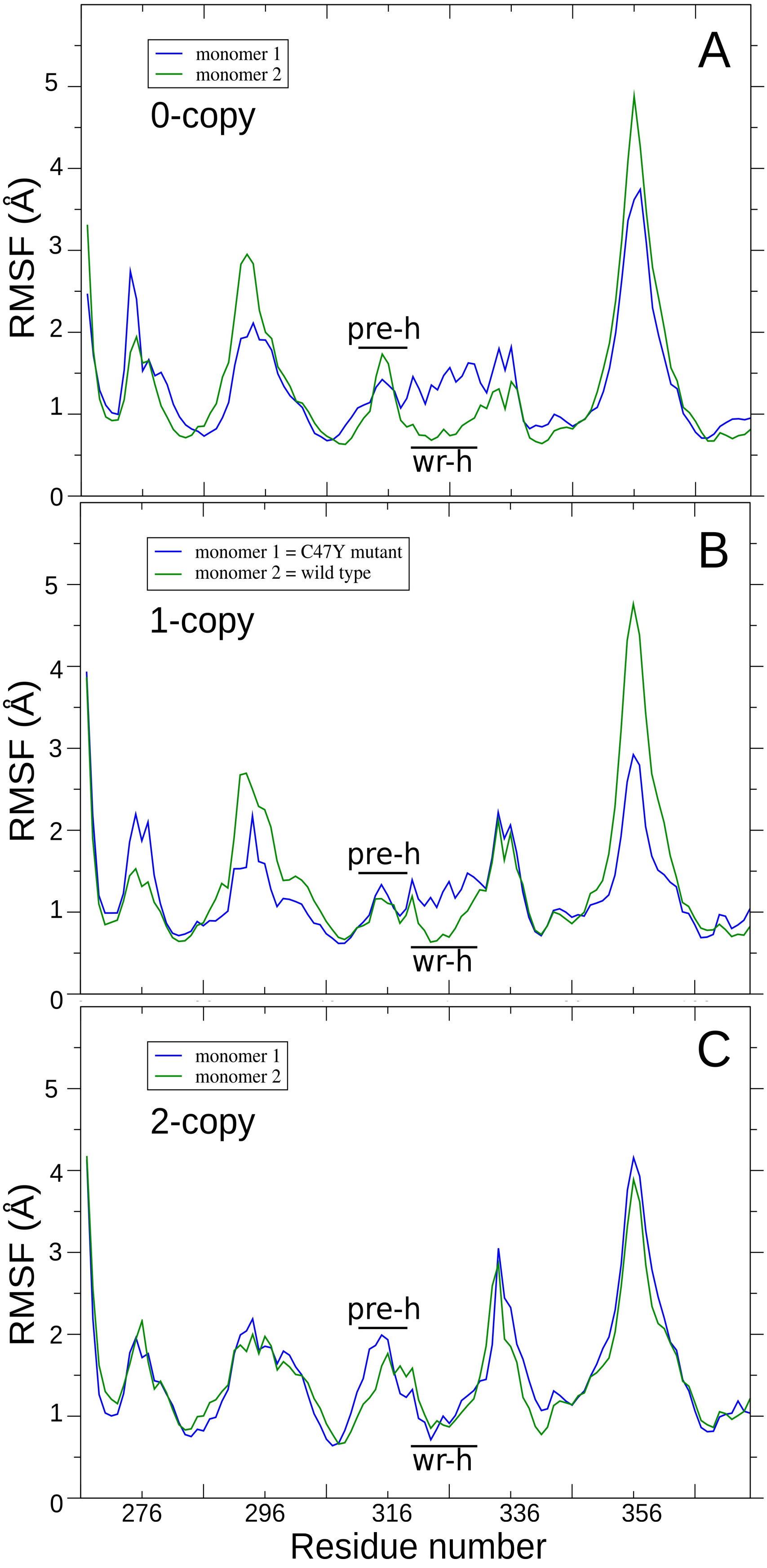


**Figure S3.** Comparison of per-residue RMSF between monomers for the 0-, 1-, and 2-copy dimers is shown in panels A-C, respectively.

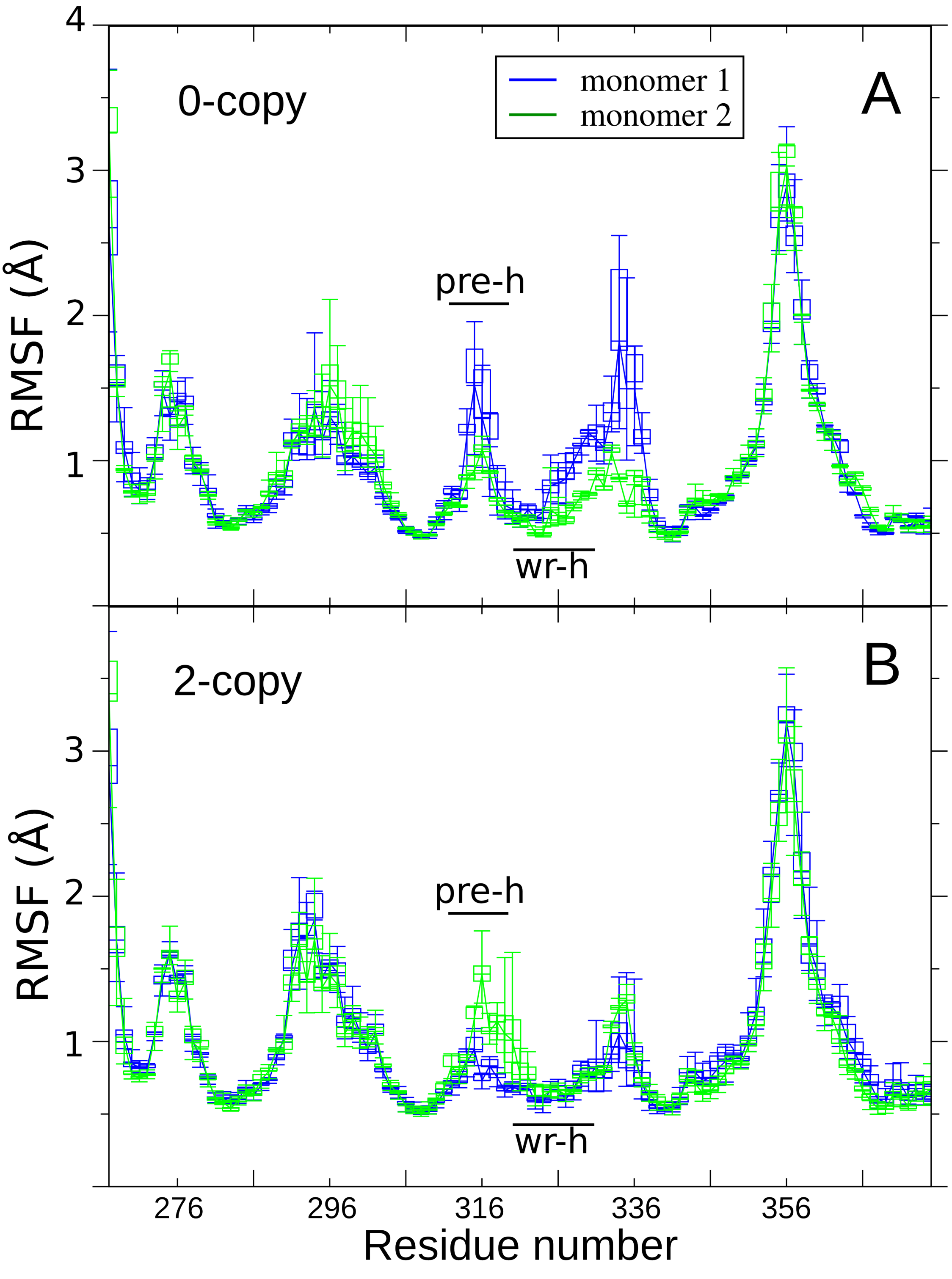


**Figure S4.** Per-residue RMSF of four time windows, each 50 ns long, shown as box-plot data for monomer 1 and 2 (blue and green colours, respectively) and for 0-copy and 2-copy (panel A and B, respectively).
